# Treatment of brucellosis in pregnant women: a systematic review and analysis of current gaps and future perspectives

**DOI:** 10.1590/S1678-9946202668032

**Published:** 2026-05-18

**Authors:** Endi Lanza Galvão, Diêgo Mendes Xavier, Glaciele Maria de Souza, Gláucia Cota, Sarah Nascimento Silva

**Affiliations:** 1 Fundação Oswaldo Cruz, Instituto René Rachou, Núcleo de Avaliação de Tecnologias em Saúde, Grupo de Pesquisa Clínica e Políticas Públicas em Doenças Infecciosas e Parasitárias, Minas Gerais, Belo Horizonte, Brazil; 2 Universidade Federal dos Vales do Jequitinhonha e Mucuri, Departamento de Fisioterapia, Programa de Pós-Graduação em Reabilitação e Desempenho Funcional, Diamantina, Minas Gerais, Brazil; 3 Universidade Federal dos Vales do Jequitinhonha e Mucuri, Programa de Pós-Graduação em Odontologia, Diamantina, Minas Gerais, Brazil

**Keywords:** Brucellosis, Pregnancy, Systematic review, Therapeutics

## Abstract

Human brucellosis is a globally prevalent bacterial disease with significant implications during pregnancy due to obstetric complications. Despite the importance of early diagnosis and treatment, the lack of therapeutic consensus and robust evidence underscores the need for systematic evaluation to guide clinical practice. This study systematically reviewed the literature to identify and summarize available drug therapy options for brucellosis in pregnant women, focusing on efficacy, safety, and obstetric outcomes. The review followed PRISMA and Cochrane Handbook guidelines. Searches were conducted in MEDLINE, Embase, Cochrane Library, LILACS, and gray literature for references indexed up to September 15, 2025. Eligible studies reported therapeutic interventions in pregnant women with brucellosis. Risk of bias was assessed using the JBI Critical Appraisal tool, and data on participants, interventions, and outcomes were extracted. Of 737 records screened, six studies met the inclusion criteria, comprising retrospective and prospective reports published between 1991 and 2020, totaling 403 pregnant women. Therapeutic regimens varied, with sulfamethoxazole–trimethoprim (SMX–TMP) plus rifampicin being the most frequently used combination. Adverse obstetric outcomes associated with this regimen occurred in 16% (95%CI: 8%–30%) of cases. Overall, abortion occurred in 94 (23.3%) women, 20 preterm births were reported, and 10 newborns had low birth weight. Recurrence was observed in only 3% of cases. Heterogeneity in outcomes and definitions limited comparability. In conclusion, there is no standardized treatment for brucellosis during pregnancy. Adverse obstetric outcomes remain frequent, highlighting the need for controlled studies to establish safe and effective therapeutic protocols.

## INTRODUCTION

Human brucellosis is a highly prevalent bacterial disease that represents a major public health issue worldwide, particularly in countries of Latin America and Sub-Saharan Africa^1^. The main clinical manifestations in adults include fever, fatigue, arthralgia, and muscle pain^2^. More severe complications, such as endocarditis and neurological, psychological, osteoarticular, and respiratory involvement, have also been reported, albeit less frequently^3^. As there are no typical clinical characteristics, diagnosis is often confused with other infections.

The presence of these nonspecific flu-like symptoms increases the risk of misdiagnosis or late diagnosis in pregnant women with brucellosis, potentially leading to irreversible consequences^4^. *Brucella* infection can result in adverse obstetric complications such as miscarriage (spontaneous or therapeutic), preterm delivery, intrauterine infection, or intrauterine fetal death (IUFD)^5^. Thus, the most relevant aspects of managing brucellosis during pregnancy are early recognition and immediate initiation of antimicrobial therapy, which may reduce the risk of adverse obstetric, neonatal, and maternal outcomes^6^.

Although the relevance of brucellosis treatment during pregnancy is well established, determining the best therapy remains a challenge. Tetracyclines are contraindicated for pregnant women, especially during the second and third trimesters, due to their potential to cause fetal tooth discoloration^7^. Administration of aminoglycosides during pregnancy increases the risk of ototoxicity or nephrotoxicity in the fetus^8^. The literature remains inconclusive regarding the safety of quinolones during pregnancy, and they are not recommended as first-line therapy, especially during the first trimester^9^.

Despite significant advances in understanding brucellosis, the lack of robust scientific evidence on the efficacy and safety of available therapeutic regimens during pregnancy represents an important gap in current medical knowledge, which remains insufficiently explored even in recent research^10^. This gap not only compromises the quality of maternal healthcare but also jeopardizes fetal well-being, highlighting the urgency of a thorough and critical analysis of the available evidence.

To our knowledge, no clinical trials have evaluated the treatment of brucellosis during pregnancy, and current therapeutic recommendations for this population are based on expert opinion, observational studies, case series, as well as clinical experience, and tradition^4^. Thus, this study aims to systematically review the scientific literature to provide an updated overview of drug therapeutic options for the primary manifestation of human brucellosis in pregnant women.

## MATERIALS AND METHODS

This systematic review was conducted in accordance with the Preferred Reporting Items for Systematic Reviews and Meta-analyses (PRISMA)^11^ and the recommendations of the Cochrane Handbook^12^. The protocol was registered in PROSPERO (CRD42023439862). The clinical question guiding the review was: “What is the efficacy and safety of antimicrobial regimens to treat human brucellosis in pregnant women?” The PICO framework (population, intervention, comparison, and outcomes) was defined as: Population – pregnant women with a confirmed diagnosis of human brucellosis; Intervention – antimicrobial monotherapy or combination therapy for the treatment of brucellosis; Comparison – antimicrobial monotherapy or combination therapy, placebo, or no treatment; Outcomes – treatment failure (represented by therapeutic failure and/or relapse), time of defervescence, treatment-related adverse events, and adverse obstetric outcomes.

### Eligibility criteria

We included original studies with any design that evaluated pharmacological interventions in pregnant women with human brucellosis and reported at least one of the outcomes of interest previously described. Only studies published in English, Spanish, or Portuguese were considered. Studies focusing on patients with secondary manifestations of human brucellosis or coinfections, as well as studies with less than five participants, were excluded. In cases of duplicate publications reporting results for the same sample, only the first publication was considered.

### Search strategy

Electronic searches were conducted in MEDLINE (via PubMed), Embase, Cochrane Library, and LILACS (via Virtual Health Library [VHL]), including records indexed up to September 15, 2025. Search strategies for each database included Mesh, Emtree, and DECS terms, as well as keywords; these are detailed in Supplementary Table S1.

Searches for gray literature were also conducted on two platforms: Google Scholar, with the main keywords defined in the search strategy, and OpenGrey (currently archived within the DANS EASY system), an European database for gray literature. Additionally, ClinicalTrials.gov, provided by the U.S. National Library of Medicine, was searched to identify registered and ongoing studies. The first 10 pages of Google Scholar were screened. For the other databases, all retrieved titles were reviewed to assess eligibility within the scope of this review.

### Study selection

Records retrieved from the databases were initially imported into the Mendeley Reference Management Software (version 1.19.8, Elsevier, Amsterdam, Netherlands) to detect and eliminate duplicates and then imported into the Rayyan platform (Rayyan Systems Inc., Cambridge, MA, USA) for reference management and study selection^13^. Two reviewers independently screened the records (titles and abstracts) and assessed full texts for eligibility. Disagreements were discussed until a consensus was reached or, when necessary, were resolved by a third reviewer.

### Data extraction and analysis

Two independent reviewers extracted and systematically recorded relevant information from each included study. This included participant characteristics (age, clinical manifestations, clinical form, duration of symptoms, *Brucella* species), methodological characteristics (year of publication, country, study design and period, inclusion criteria, diagnostic criteria, length of follow-up), intervention characteristics (treatment regimen, dosage, and duration), and outcomes. If relevant information was missing from the primary studies, the authors planned to contact study investigators via email; however, this was not necessary, as all required data were available in the published articles.

A narrative summary of the findings was conducted, structured around key themes to deepen the understanding of the results and to identify underlying concepts and patterns. The pooled prevalence was estimated using a random-effects meta-analysis of single proportions with inverse-variance weighting after logit transformation of proportions (sm = ‘PLOGIT’ in meta::metaprop), with pooled estimates back-transformed to the proportion scale for presentation. In this analysis, only therapeutic regimens used in five or more patients were included, and subgroup analyses by treatment were performed to facilitate interpretation of the results. All data were organized and analyzed using R statistical software (version 4.4.0, R Foundation for Statistical Computing, Vienna, Austria), employing the meta package for conducting and visualizing meta-analyses.

### Assessment of risk of bias

Risk of bias was assessed using the Joanna Briggs Institute (JBI) Critical Appraisal Checklist for Case Series, considering the descriptive nature of the studies^14^. Each item on the checklist was rated as “yes,” “no,” or “unclear” based on the information reported in the studies to determine their methodological quality.

## RESULTS

**Figure 1:**
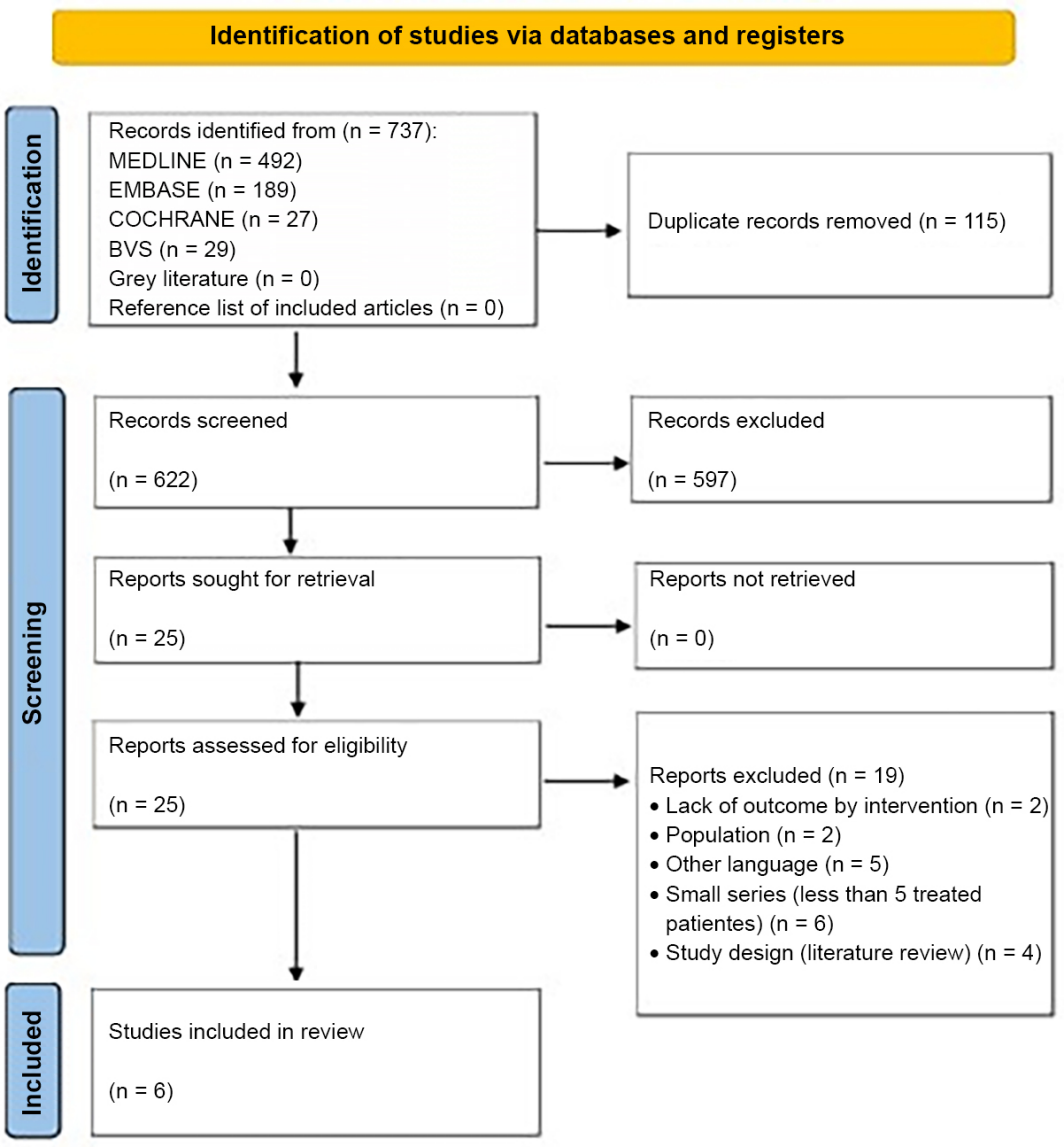
PRISMA flow diagram.

The search identified 737 records across the databases. After screening and selection based on eligibility criteria, six studies were included in the descriptive analysis^15-20^. The study selection process and reasons for exclusion are summarized in Figure 1. More detailed information about excluded studies is presented in Supplementary Table S2.

Of the included studies, four were retrospective reports on the treatment of human brucellosis in pregnant women^15-17,20^ and two were prospective studies^18,19^, published between 1991 and 2020. The studies were conducted in the Republic of Macedonia^15^, Saudi Arabia^20^, Turkey^17,18^, Iran^19^, and Lebanon^16^. A total of 403 pregnant women were included, ranging from five to 242 participants. The characteristics of the eligible studies, including methodology, participants, and intervention details, are summarized in Tables ,2 and 3. Table 4 details dosages and posology of each antimicrobial used in the treatments of pregnant women with brucellosis. These tables enable a qualitative comparison across studies, considering differences in study design, clinical presentation, diagnostic criteria, antimicrobial regimens, and obstetric outcomes. This structured qualitative synthesis highlights the clinical and methodological heterogeneity of the available evidence and supports the interpretation of the quantitative findings.

**Table 1:** Main methodological characteristics of studies on the treatment of brucellosis in pregnant women

Article	Country (n)	Study design	Study period	Follow-up	Diagnostic criteria	Inclusion criteria
Bosilkovski *et al*.^15^	Republic of Macedonia (5)	Case series	1995–2009	6–36 months	Clinical signs and symptoms, laboratory analyses (Hb 120 g/L, RBC 4100x1012/L, leukocytes 4.6x109/L, Ne 62%, Pl 200x1012/L, ALT/AST 22/18 U/L, creatinine 64 μmol/L, ESR 60 mm/h, PCR 64 mg/L, negative urine culture, positive Rose Bengal, STA 1/1280, Brucella Coombs test 1/1280)	None of the patients had a previous history of brucellosis, chronic venereal diseases, pathological pregnancies, or miscarriages
Inan *et al*.^17^	Türkiye (242)	Multicenter retrospective observational	2002–2015	Pregnancy + neonatal period and after therapy completion	Clinical and laboratory confirmation, STA (≥ 1:160) or RBT and/or *Brucella* isolation in human blood and/or placenta and aborted fetus	Pregnant women with brucellosis and with/without recent birth/abortion/DFI and women with known brucellosis who became pregnant and with/without recent birth/abortion/DFI were the inclusion criteria
Gulsun *et al*.^18^	Türkiye (39)	Retrospective observational	2003–2010	Pregnancy + neonatal period and for at least six months after therapy completion	STA test, Coombs test anti-Brucella and/or blood culture system in pregnant women whose clinical findings suggested brucellosis	Healthy pregnant women as a control group and pregnant women who had brucellosis
Roushan *et al*.^19^	Iran (19)	Case series	2000–2010	24 months	STA titer ≥1/160 and 2-Mercaptoethanol (2ME) titer ≥1/ 80 with compatible clinical findings	NR
Khan *et al*.^20^	Saudi Arabia (92)	Retrospective observational	1983–1995	NR	Compatible clinical features and a serum agglutinin titer of ≥1:320 or a positive blood culture result	NR
Seoud *et al*.^16^	Lebanon (6)	Case series	1985–1987	Eight months postpartum	Clinical signs and symptoms, serological test using the direct and indirect agglutination tube test (direct titer >1:80 and indirect titer >1:160). All cultures were performed in special media	Pregnant women with brucellosis

n *=* number of participants; NR = not reported; Hb = hemoglobin; RBC = red blood cells; Ne = neutrophils; Pl = platelets; ALT = alanine aminotransferase; AST = aspartate aminotransferase; U/L = units per liter; μmol/L = micromoles per liter; ESR = erythrocyte sedimentation rate; mm/h = millimeters per hour; CRP = C-reactive protein; mg/L = milligrams per liter; PCR = polymerase chain reaction; STA = standard tube agglutination test; RBT = Rose Bengal test; 2ME = 2-mercaptoethanol test; DFI = fetal death in utero.

**Table 2:** Characteristics of the population included in studies on the treatment of human brucellosis during pregnancy.

Article	Age mean±SD (range) years	Gestational age at onset (Weeks/Trimester)	Clinical manifestation (%)	Clinical form	Brucella species	Duration of symptoms
Bosilkovski *et al*.^15^	25.8 ± 3.2 (20–29)	Variable (weeks 13–39)	Case 1 (low back pain, malaise, sweating, headache, and temperature of 38.4 °C). Case 2 (temperature of 39 °C, fever, malaise, arthralgias, sweating). Case 3 (malaise, pain in the right hip, temperature of 39 °C, night sweats, decreased appetite, and headache). Case 4 (sweating, malaise, headache, decreased appetite, arthralgias, nausea, and vomiting for three weeks). Case 5 (temperature of 38.2 °C, sweating, malaise, headache, arthralgia, and left hip arthritis).	NR	NR	Case 1: two weeks until the diagnosis of brucellosis. Case 2: Six weeks before hospital admission Case 3: Two months before hospital admission Case 4: 3 weeks Case 5: Three months before his admission to the hospital
Inan *et al*.^17^	28.8 ± 6.28 (17–50)	NR	Weakness (95.4%), night sweats (89.6%), arthralgia (88.8%), fever (83.7%), vaginal bleeding (9.1%), groin pain (23.5% ).	Acute: 230 (95.0%) Subacute: 7 (2.9%) Chronic: 5 (2.1%)	Brucella spp. (41.8%) B. melitensis (64.2%)	Average of 15 (range 10–21) days
Gulsun *et al*.^18^	28.07 (20–40)	NR	Arthralgia (92.3%), fever (76.9%), myalgia (71.7%), hepatomegaly (28.2%), and splenomegaly (25.6%).	Acute: 30 Subacute: 2 Recurrence: 4	B. melitensis (NR)	NR
Roushan *et al*.^19^	25.0 ± 4.62	NR	Sweating (57.8%), fever (42.1%), back or generalized pain (36.8%), arthralgia, or myalgia (36.8%)	NR	Brucella melitensis (4)	NR
Khan *et al*.^20^	26.9 (16–47)	NR	Fever (41.3%)	Acute: 92 (100%)	Brucella melitensis (16) Brucella abortus (1) Not identified (5)	NR
Seoud *et al*.^16^	24.5 (18 – 33)	First Quarter (n=2/ NR)	Fever (100%), generalized malaise (83.3%), fatigue (83.3%), chills (66.6%), sweating (66.6%), low back pain (66.6%), arthralgia (50%), myalgia (50%), headache (33.3%), hepatosplenomegaly (33.3%)	NR	B. melitensis (3)	Fever – Average 3.5 (±3.0) (range 2 - 12 weeks)

SD = standard deviation; NR = not reported; *spp. =* species pluralis; *Brucella spp. =* species of the genus *Brucella*; *B.*, *Brucella*; n = number of cases; mean ± SD = mean and standard deviation; range = minimum and maximum values; weeks = gestational weeks; trimester (quarter) = gestational trimester; acute = acute clinical form; subacute = subacute clinical form; chronic = chronic clinical form.

**Table 3:** Results of brucellosis treatment in pregnant women from studies included in the systematic review

Article	Interventions (n)	Duration of treatment (days)	Follow-up (months)	Clinical evolution (n/N treated)	Definition of recurrence	Obstetric outcomes (n/N treated)
Bosilkovski *et al.* ^15^	SMX-TMP + RF + DX (1) **	28	36	Recurrence (1/1)	NR	Vaginal bleeding and miscarriage in the 13^th^ week of gestation (during treatment with SMX-TMP + RF) (1/1)
	SMX-TMP + RF (1)	45	12	Defervescence on the second day of treatment (1/1)	NR	Absence of movements fetalis in the 28^th^ week of pregnancy (3 months after antibrucellar treatment had been finished) (1/1)
	SMX-TMP + RF + DX (1) **	28	19	Defervescence on the third day of treatment, cure (1/1)	NR	Premature birth in the 32^nd^ gestational week without symptoms/signs and negative Brucellacapt (1/1)
	SMX-TMP + RF (1)	45	12	Cure without recurrence (1/1)	NR	Premature birth in the 32^nd^ gestational week, six weeks after completion of the antibrucellar treatment. Initial positive Rose Bengal in babies and negative after 9 months of birth (1/1)
	SMX-TMP + RF (1)	45	6	Defervescence on the fourth day of treatment, cure without recurrence (1/1)	NR	Spontaneous vaginal birth in the 39^th^ week of pregnancy, three weeks after the end of antibrucellar treatment. The baby showed positive results in serological tests for the first 6 months, after which they were negative (1/1)
Inan *et al*.^17^	CFX + RF (92) SMX-TMP + RF (79) SMX-TMP + CFX + RF (47) SMX-TMP + GT/STP (6) CFX + SMX-TMP (3) RF + DX (3) RF + SMX-TMP + GT (3) RF + GT (1) RF+DX+GT (1) CFX (5) CFM (2)	42–56 (or until resolution)	NR	Recurrence DX + RF (1/ 3)*	Reappearance of clinical signs and of brucellosis after successful therapy and a positive culture or a rise in antibody titer in the absence of symptoms of exposure to Brucellae.	Obstetric complications b (CFX + RF 12/92; SMX-TMP + RF 11/79; SMX-TMP + CFX + RF 4/47) Maternal and newborn mortality or pertinent sequelae in the newborns (0/242)
Gulsun *et al.* ^18^	SMX-TMP (NR) SMX-TMP + RF (NR) CFX + RF (NR) RF (NR)	42	6	Recurrence SMX-TMP (7/NR)	Previously diagnosed with brucellosis and cured appropriately but clinical and laboratory findings had again become positive.	Full-term births (16), low birth weight (10), pre-term births (7), abortion in the first trimester (1)***
Roushan *et al*.^19^	SMX-TMP + RF (13) GT + DX (4) SMX-TMP + DX (1) DX + RF (1)	45 to 60	24	NR	NR	Term deliveries (SMX-TMP + RF 8/13) Spontaneous abortion (SMX-TMP + RF 5/13; GT + DX 4/4; SMX-TMP + DX 1/1; DX + RF 1/1)
Khan *et al*.^20^	SMX-TMP (23) SMX-TMP + RF (17) RF (1)	At least 4 weeks	NR	NR	NR	Spontaneous abortion or fetal death (SMX-TMP 3/23; SMX-TMP + RF 1/17; RF 0/1) Normal deliveries (SMX-TMP 19/23; SMX-TMP + RF 16/17; RF 1/1)
Seoud *et al*.^16^	SMX-TMP + CFT (1)	42	NR	Recurrence (1/1)	NR	A therapeutic abortion in the 10th week of pregnancy (1/1)
	SMX-TMP + RF (2)	35–42	NR	Recurrence (0/2)	NR	Normal vaginal delivery (1/1)
	NR	NR	NR	Recurrence (0/1)	NR	Premature rupture of membranes, chorioamnionitis, cesarean section (1/1)
	SMX-TMP + TE (2)	21 to 28	NR	Recurrence (0/2)	NR	Therapeutic abortion in the 12th week of pregnancy (1/1)

n *=* number of patients; N = total number treated; NR = not reported; SMX-TMP = trimethoprim–sulfamethoxazole; RF = rifampicin; DX = doxycycline; CFX = ciprofloxacin; CFM = cefuroxime; CFT = ceftriaxone; GT = gentamicin; STP = streptomycin; TE = tetracycline; days = duration of treatment in days; months = follow-up period in months; defervescence = resolution of fever; recurrence = reappearance of clinical and/or laboratory evidence of brucellosis after completion of adequate therapy; Brucellacapt = immunocapture agglutination test for brucellosis; *overall results without distinction by intervention; **added doxycycline to treatment after miscarriage or birth of baby; ***the study does not present the outcomes per intervention.

**Table 4:** Dosage of therapies used in pregnant women from studies included in the systematic review

Article	Interventions/Dosages							
	SMX-TMP	RF	DX	CFX	CFM	GT	CFT	TE
Bosilkovski *et al*.^15^	NR	NR	NR	NR	-	-	-	-
Inan *et al*.^17^	NR	NR	NR	NR	NR	NR	-	-
Gulsun *et al*.^18^	80 mg of Trimethoprim (TMP) and 400 mg of sulfamethoxazole (SMX) every 12 h*	600 mg/day by mouth for six weeks	-	2 g/day/iv	-	-	-	-
Roushan *et al*.^19^	Cotrimoxazole 8 mg/kg/day by component of trimethoprim in three divided daily doses and dosage	15 mg/kg/day once a day in the morning before breakfast	100 mg twice a day	-	-	5 mg/kg/day	-	-
Khan *et al*.^20^	Cotrimoxazole as one double strength tablet (800 mg or sulfamethoxazole and 160 mg of trimethoprim), orally b.i.d.	600–900 mg, orally q.d.	-	-	-	-	-	-
Seoud *et al.* ^16^	2 tablets/day for three to six weeks	600 mg/day for six weeks	-	-	-	-	4 /day for three days	2 g/day for two or three weeks

SMX-TMP = trimethoprim–sulfamethoxazole (cotrimoxazole); RF = rifampicin; DX = doxycycline; CFX = ciprofloxacin; CFM = cefuroxime; GT = gentamicin; CFT = ceftriaxone; TE = tetracycline; NR = not reported; iv = intravenous; orally/by mouth = oral administration; day = per day; b.i.d. = twice daily; q.d. = once daily; *TMP-SMX was not administered in the third trimester because of the risk of jaundice and kernicterus in the fetus.

Although a total of 403 pregnant women with brucellosis were included across the studies, obstetric outcomes were explicitly reported and extractable for only 179 women. This limitation arose because several studies, particularly those with larger samples, presented outcome data in an aggregated manner or did not provide sufficient detail for individual outcome extraction.

The studies reported the use of different therapeutic regimens, ranging from monotherapy to dual and triple therapy. The most commonly used regimen was the combination of sulfamethoxazole–trimethoprim (SMX–TMP) + rifampicin, prescribed for 114 pregnant women^15-17,19,20^. This was followed by ceftriaxone + rifampicin, prescribed for 92 pregnant women, although this regimen was evaluated in only one study^17^. Bosilkovski *et al*.^15^ reported the addition of doxycycline to the SMX–TMP + rifampicin regimen after abortion or delivery—this approach was not mentioned in the other studies. The triple regimen of SMX–TMP + ceftriaxone + rifampicin was prescribed for 47 pregnant women^17^. Disease recurrence was reported in four studies, occurring in 10 participants (3%) after treatment and resolution of clinical signs and symptoms^15-18^. However, not all studies linked recurrence to the specific therapeutic regimen used. Time to defervescence was reported in only one study^15^, ranging from two to four days after treatment with SMX–TMP + rifampicin. No studies reported detailed safety profiles of the therapeutic regimens in the pregnant women evaluated. Inan *et al*.^17^ found no significant difference in the rates of adverse obstetric outcomes among pregnant women treated with SMX–TMP + rifampicin and those treated with ceftriaxone + rifampicin (13.9% versus 13.0%, p = 0.849).

It was not possible to compare the performance of different therapeutic regimens in the same study, since results were described in an aggregated manner^17^, detailed for only a few participant groups^18^, or limited to a single participant per regimen^15,16^.

Pregnancy-related outcomes were reported in all studies. Abortion (spontaneous or therapeutic) was observed in all studies, affecting 94 pregnant women (23.3%). A total of 20 preterm births were reported^17,18^, and 10 newborns presented low birth weight^18^. Full-term births were reported in 65 newborns^18-20^.

In the study by Khan *et al*.^20^, 92 pregnant women were included, of whom 41 received antimicrobial treatment for brucellosis. Prenatal treatment had a significant protective effect against abortion when compared with inadequate or no treatment (RR: 0.14; 95%CI: 0.06–0.37; *p* > 0.001). However, the incidence of miscarriage among the 23 patients treated with SMX-TMP monotherapy did not differ significantly from that observed in the 17 patients treated with SMX–TMP + rifampicin (*p* = 0.6).

**Figure 2:**
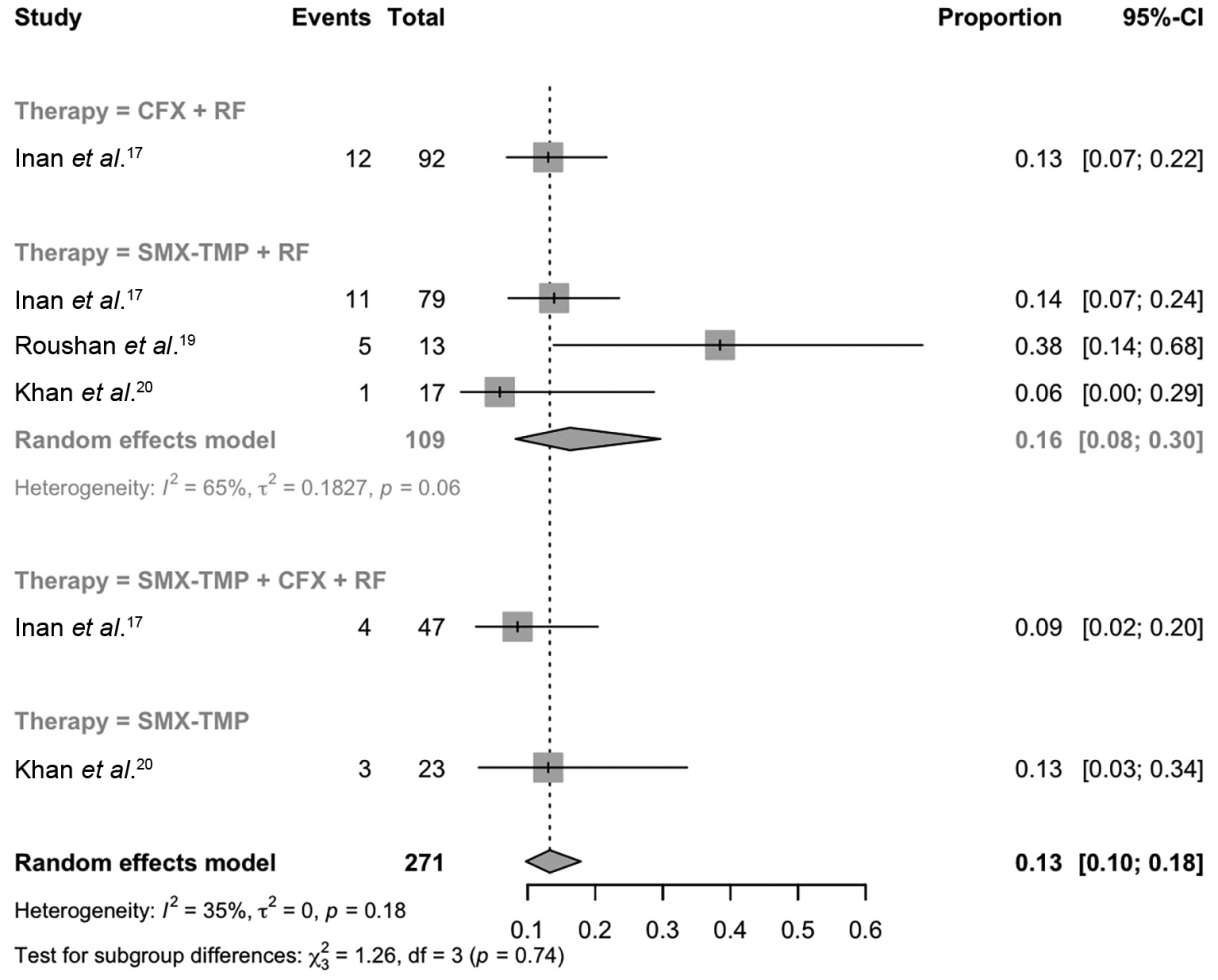
Pooled rate of adverse obstetric events associated with therapeutic approaches for brucellosis in pregnant woman.

Although heterogeneity in outcomes, definitions, and study designs precluded groupings and comparisons for most outcomes of interest, it was possible to estimate the rate of adverse obstetric outcomes, especially for SMX-TMP + rifampicin, which was 16% (95%CI: 0.08–0.30, *I* ^2^ = 36%, τ^2^ = 0.1827, *p* = 0.06, Figure 2). For the other regimens, the estimates are based on a single study.

As the studies included in this review were observational, did not aim to compare interventions, and evaluated prevalent cases in convenience samples of patients with similar characteristics, we considered it appropriate to classify all of them as case series^14^. Thus, scores on the JBI Critical Appraisal Checklist for Case Series (maximum of 10 points) ranged from 6 to 10 points (Supplementary Figure S1). Gulsun *et al.* ^18^ showed low risk of bias across all domains evaluated by the tool, whereas Seoud *et al*.^16^ had a high risk of bias in the reporting of outcomes, which was unclear. Roushan *et al*.^19^ and Khan *et al*.^20^ did not clearly report whether participants were consecutively included. Additionally, Khan *et al*.^20^ did not report clinical signs and symptoms, limiting the evaluation of the domain related to clarity of clinical and demographic information. Overall, several domains were rated as unclear due to insufficient reporting, particularly those related to standardized and reliable measurements, consecutive inclusion of participants, and presentation of demographic information of the study population.

## DISCUSSION

This systematic review of the literature on the treatment of brucellosis in pregnant women revealed several challenges and complexities associated with this clinical condition. The descriptive analysis of the six included studies provided an updated overview of current therapeutic practices, observed results, and pregnancy-related outcomes. In this discussion, we highlight the key findings and their clinical and research implications.

First, the diversity of therapeutic regimens across studies highlights the lack of consensus in the management of brucellosis in pregnant women. The variation between mono, dual, and triple therapy, with different antibiotic combinations, highlights the urgent need for standardized therapeutic protocols. The predominance of combined therapy—especially SMX–TMP + rifampicin in the studies that evaluated the largest numbers of pregnant women^18,21^—along with the absence of reported recurrence associated with these regimens, suggests a possible therapeutic benefit, as observed in adult populations^22^. However, data regarding pregnant women remain insufficient to draw firm conclusions. Moreover, inconsistent and imprecise reporting of pregnancy-related adverse outcomes further limits the ability to draw definitive conclusions regarding this combination.

The relapse rate of the disease raises concerns about the long-term effectiveness of the therapeutic regimens adopted. Although some included studies reported relapse in pregnant women^16-19^, this outcome has not been consistently described in relation to specific interventions within standardized protocols. The lack of uniformity in the definition and evaluation of outcomes across studies limits direct comparison and generalizability, emphasizing the need for standardized criteria to assess recurrence.

The analysis of pregnancy-related outcomes indicates the occurrence of adverse obstetric events, although the available evidence is heterogeneous and limited. Abortion was a frequent outcome across studies, affecting 8% of pregnant women, while the threat of abortion was reported in 2%. Intrauterine fetal death was also observed, along with preterm birth and low birth weight. Only one study was adequately designed to assess whether adverse obstetric events were associated with the natural history of the disease or with treatment^21^. This study compared the occurrence of abortion between patients who received and did not receive prenatal antimicrobial therapy, suggesting a lower incidence of abortion in the treated group^21^. However, no differences were found in abortion rates between therapeutic regimens. Another study included a control group of pregnant women without brucellosis^19^ and reported a significantly higher rate of preterm birth among women with brucellosis compared to controls (17.9% versus 2.5%, p = 0.01). Similarly, the risk of low birth weight was higher in the brucellosis group. However, this study did not assess associations with specific treatments or control for confounding variables. Overall, these findings emphasize the vulnerability of pregnant women with brucellosis and underscore the need for safer and more effective therapeutic strategies to preserve maternal and fetal health.

In addition to transmission via contact with infected animals^23^, consumption of unpasteurized dairy products^24^, and accidental exposure^25^, brucellosis is also a health condition that can be transmitted between humans via the placental barrier and lactation^4,26^. Although some literature reviews on brucellosis during pregnancy have been published^4,26-28^, none have systematically investigated the use of antibiotic therapy in pregnant women with brucellosis. A recently published scoping review addressing treatments in the context of maternal and child health did not provide detailed descriptions of the included studies and regimens, particularly regarding effectiveness and safety outcomes^10^. In general, while some studies have discussed treatment approaches, such as the use of rifampicin as an alternative to tetracyclines^4,6,26,27^, important gaps remain regarding the choice of drugs, dosing, and treatment duration, which motivated this review. However, the lack of robust clinical studies evaluating the efficacy and safety of different treatment regimens in pregnant women continues to limit the broader understanding of this condition during pregnancy.

Despite the lack of robust evidence, establishing treatment recommendations for this population remains essential in clinical practice. We observed that the World Health Organization (WHO)^28^ recommends treatment with rifampicin for six weeks, which may be combined with SMX–TMP, since tetracyclines are contraindicated. These drugs are categorized as C according to the U.S. Food and Drug Administration (FDA) pregnancy risk classification, indicating that their use requires caution and monitoring during pregnancy^29,30^. Furthermore, SMX–TMP should be avoided after 36 weeks of gestation due to the risk of kernicterus caused by elevated bilirubin levels^31^.

In addition to drug treatment, health education aimed at pregnant women and women of reproductive age is a crucial factor for disease management. Knowledge of the disease, forms of acquisition, clinical manifestations, and potential consequences if untreated contribute to prevention, timely diagnosis, and early treatment. Despite its importance, none of the included studies addressed this issue. Such actions should be incorporated into public health policies, especially in endemic areas^6^. A multidisciplinary approach and collaboration among healthcare professionals are also fundamental for developing solid guidelines aimed at ensuring the safety of both mother and fetus during the treatment of brucellosis during pregnancy.

High-quality evidence to guide the treatment of human brucellosis remains limited, largely because the disease is neglected and because randomized clinical trials in this population pose ethical and practical challenges. Consequently, observational designs—such as cohort studies and large case series—are essential to elucidate therapeutic effectiveness. Although several such studies were identified in this review, many were excluded because they did not report outcomes stratified by treatment regimen^32,33^ or consisted of very small case reports/series (<5 patients)^34-38^. The latter was an a priori exclusion criterion to mitigate the high risk of bias associated with extremely small samples. Reports focusing on secondary manifestations of human brucellosis, while clinically informative, were also outside the scope of this review, which centered on standard therapeutic regimens for the general patient population.

The small number of eligible studies and their inherent risk of bias represent the main limitations of this review. Substantial methodological heterogeneity, including differences in study design, diagnostic criteria, therapeutic regimens, follow-up duration, and outcome definitions, limits comparability across studies. The predominance of retrospective designs and case series increases the risk of selection bias, while inconsistencies in outcome definitions further constrain the robustness of pooled estimates. Consequently, findings from the meta-analysis of proportions should be interpreted with caution.

The findings of this systematic review underscore the urgent need for further research, particularly well-designed prospective observational studies and controlled clinical trials, to establish specific and effective therapeutic protocols for pregnant women with brucellosis. Standardization of diagnostic criteria, outcome definitions, and therapeutic regimens are essential to improve comparability across studies and enhance the generalizability of findings, ultimately supporting a more evidence-based and safer clinical approach to this challenging condition.

## CONCLUSIONS

The review highlights the lack of consensus on therapeutic regimens for brucellosis in pregnant women, indicating the need for standardized protocols. Adverse obstetric outcomes were frequent, emphasizing the vulnerability of this population. The scarcity of controlled studies and the risk of bias in the included studies emphasize the importance of further clinical research and more consistent case reports to guide an effective and safe approach to this challenging condition during pregnancy.

## Supplementary Materials

SUPPLEMENTARY MATERIAL

## Data Availability

The complete anonymized dataset supporting the findings of this study is available from https://doi.org/10.48331/SCIELODATA.FOLHYO
