# Supplementary material for: Treatment of brucellosis in pregnant women: a systematic review and analysis of current gaps and future perspectives
Source: Rev Inst Med Trop Sao Paulo. 2026 May 18;68:e32. doi: 10.1590/S1678-9946202668032 (PMC13185525; doi:10.1590/S1678-9946202668032)
Supplement: SUPPLEMENTARY MATERIAL [file 1678-9946-rimtsp-68-S1678-9946202668032-suppl01.pdf]

REVISTA DO INSTITUTO DE MEDICINA TROPICAL DE SÃO PAULO

JOURNAL OF THE SÃO PAULO INSTITUTE OF TROPICAL MEDICINE

SUPPLEMENTARY MATERIAL

https://doi.org/10.48331/SCIELODATA.FOLHYO

Treatment of brucellosis in pregnant women: a systematic review and analysis of current gaps and future perspectives

Endi Lanza Galvão [1,2], **Diêgo Mendes Xavier** [2], **Glaciele Maria de Souza** [3], **Gláucia Cota** [1], **Sarah Nascimento Silva** [1]

[1]Fundação Oswaldo Cruz, Instituto René Rachou, Núcleo de Avaliação de Tecnologias em Saúde, Grupo de Pesquisa Clínica e Políticas Públicas em Doenças Infecciosas e Parasitárias, Minas Gerais, Belo Horizonte, Brazil

[2]Universidade Federal dos Vales do Jequitinhonha e Mucuri, Departamento de Fisioterapia, Programa de Pós-Graduação em Reabilitação e Desempenho Funcional, Diamantina, Minas Gerais, Brazil

[3]Universidade Federal dos Vales do Jequitinhonha e Mucuri, Programa de Pós-Graduação em Odontologia, Diamantina, Minas Gerais, Brazil

Correspondence to: Diego Mendes Xavier
Universidade Federal dos Vales do Jequitinhonha e Mucuri, Departamento de Fisioterapia, Programa de Pós-Graduação em Reabilitação e Desempenho Funcional, Rua da Glória, 187, CEP 39100-000, Diamantina, MG, Brazil
Tel: +55 38 3532-6000
E-mail: diego.mendes@ufvjm.edu.br

Received: 22 November 2025
Accepted: 12 March 2026

Editor: Thelma Suely Okay

	Bosilkovski, 2020	Inan, 2019	Gulsun, 2011	Roushan *et al.*, 2011	Khan *et al.*, 2001	Seoud, 1991
1. Were there clear criteria for inclusion in the case series?	Y	Y	Y	N	N	Y
2. Was the condition measured in a standard, reliable way for all participants included in the case series?	U	U	Y	Y	Y	Y
3. Were valid methods used for identification of the condition for all participants included in the case series?	Y	Y	Y	Y	Y	Y
4. Did the case series have consecutive inclusion of participants?	U	Y	Y	Y	Y	U
5. Did the case series have complete inclusion of participants?	Y	Y	Y	Y	Y	Y
6. Was there clear reporting of the demographics of the participants in the study?	Y	Y	Y	Y	Y	Y
7. Was there clear reporting of clinical information of the participants?	Y	Y	Y	Y	Y	Y
8. Were the outcomes or follow up results of cases clearly reported?	Y	Y	Y	Y	Y	N
9. Was there clear reporting of the presenting site(s)/clinic(s) demographic information?	U	Y	Y	Y	N	Y
10. Was statistical analysis appropriate?	NA	Y	Y	NA	Y	Y

Supplementary Figure S1 - Risk of bias assessment by *JBI Critical Appraisal Checklist for Case Series.* Legend: Y = Yes, N = No, U = Unclear, NA = Not applicable

This is an open-access article distributed under the terms of the Creative Commons Attribution License.

Supplementary Table S1 - Search strategy

Database	Search strategy	*Hits + update*
**MEDLINE (via Pubmed)**	**Population:**<br>**#1** (Brucellosis[MeSH Terms]) OR (Brucellosis) OR (Brucelloses) OR (Malta Fever) OR (Fever, Malta) OR (Gibraltar Fever) OR (Fever, Gibraltar) OR (Rock Fever) OR (Fever, Rock) OR (Cyprus Fever) OR (Fever, Cyprus) OR (Brucella Infection) OR (Brucella Infections) OR (Infection, Brucella) OR (Undulant Fever) OR (Fever, Undulant) OR (Brucella abortus) OR (Brucella canis) OR (Brucella melitensis) OR (Brucella ovis) OR (Brucella suis)<br>**Population:**<br>**#2** "pregnan*"[All Fields]<br>**Intervention:**<br>**#3** (therapeutics[MeSH Terms] OR antibacterial agents[MeSH Terms]) OR (antibiotics[MeSH Terms]) OR (Therapeutics) OR (Therapeutic) OR (Therapy) OR (Therapies) OR (Treatment) OR (Treatments) OR (Anti-Bacterial Agents) OR (Agents, Anti-Bacterial) OR (Anti Bacterial Agents) OR (Antibacterial Agents) OR (Agents, Antibacterial) OR (Antibacterial Agent) OR (Agent, Antibacterial) OR (Anti-Bacterial Compounds) OR (Anti Bacterial Compounds) OR (Compounds, Anti-Bacterial) OR (Anti-Bacterial Agent) OR (Agent, Anti-Bacterial) OR (Anti Bacterial Agent) OR (Anti-Bacterial Compound) OR (Anti Bacterial Compound) OR (Compound, Anti-Bacterial) OR (Bacteriocidal Agents) OR (Agents, Bacteriocidal) OR (Bacteriocidal Agent) OR (Agent, Bacteriocidal) OR (Bacteriocide) OR (Bacteriocides) OR (Anti-Mycobacterial Agents) OR (Agents, Anti-Mycobacterial) OR (Anti Mycobacterial Agents) OR (Anti-Mycobacterial Agent) OR (Agent, Anti-Mycobacterial) OR (Anti Mycobacterial Agent) OR (Antimycobacterial Agent) OR (Agent, Antimycobacterial) OR (Antimycobacterial Agents) OR (Agents, Antimycobacterial) OR (Antibiotics) OR (Antibiotic)<br>**#4** #1 AND #2 AND #3	492
**EMBASE**	**Population:**<br>**#1** ('brucellosis'/exp OR 'brucella infection' OR 'brucella melitensis infection' OR 'malta fever' OR 'mediterranean fever (brucellosis)' OR 'brucellosis' OR 'infection by brucella' OR 'infection by brucella melitensis' OR 'infection due to brucella' OR 'infection due to brucella melitensis' OR 'melitococcosis' OR 'undulant fever' OR 'brucella'/exp OR 'brucella' OR 'brucella contamination' OR 'brucella abortus'/exp OR 'brucella abortus' OR 'bacterium abortus' OR 'brucella abortus bang bacteria' OR 'brucella abortus sensitivity' OR 'brucella canis'/exp OR 'brucella melitensis'/exp OR 'brucella melitensis' OR 'micrococcus melitensis' OR 'brucella ovis'/exp OR 'brucella ovis' OR 'brucella suis'/exp OR 'brucella melitensis biovar suis' OR 'brucella melitensisbv. suis' OR 'brucella suis')<br>**Population:**<br>**#2** pregnan*<br>**Intervention:**<br>**#3** 'therapy' OR 'combination therapy' OR 'disease therapy' OR 'disease treatment' OR 'diseases treatment' OR 'disorder treatment' OR 'disorders treatment' OR 'efficacy, therapeutic' OR 'illness treatment' OR 'medical therapy' OR 'medical treatment' OR 'multiple therapy' OR 'polytherapy' OR 'somatotherapy' OR 'therapeutic action' OR 'therapeutic efficacy' OR 'therapeutic trial' OR 'therapeutic trials' OR 'therapeutics' OR 'therapy, medical' OR 'treatment effectiveness' OR 'treatment efficacy' OR 'treatment, medical'<br>**#4** #1 AND #2 AND #3 AND [embase]/lim	189

Supplementary Table S1 - Search strategy (cont.)

Database	Search strategy	*Hits + update*
**Cochrane Library**	**Population:**<br>**#1** MeSH descriptor: [Brucellosis] explode all trees<br>**Population:**<br>**#2** 'brucellosis' OR 'brucella infection' OR 'brucella melitensis infection' OR 'malta fever' OR 'mediterranean fever (brucellosis)' OR 'brucellosis' OR 'infection by brucella' OR 'infection by brucella melitensis' OR 'infection due to brucella' OR 'infection due to brucella melitensis' OR 'melitococcosis' OR 'undulant fever' OR 'brucella' OR 'brucella abortus' OR 'brucella abortus' OR 'bacterium abortus' OR 'brucella abortus bang bacteria' OR 'brucella abortus sensitivity' OR 'brucella melitensis' OR 'brucella canis' OR 'brucella canis' OR 'brucella canidis' OR 'brucella ovis' OR 'brucella ovis' OR 'brucella suis' OR 'brucella melitensis biovar suis' OR 'brucella melitensisbv. suis' OR 'brucella suis"brucellosis' OR 'brucella infection' OR 'brucella melitensis infection' OR 'malta fever' OR 'mediterranean fever (brucellosis)' OR 'brucellosis' OR 'infection by brucella' OR 'infection by brucella melitensis' OR 'infection due to brucella' OR 'infection due to brucella melitensis' OR 'melitococcosis' OR 'undulant fever' OR 'brucella' OR 'brucella abortus' OR 'brucella abortus' OR 'bacterium abortus' OR 'brucella abortus bang bacteria' OR 'brucella abortus sensitivity' OR 'brucella melitensis' OR 'brucella canis' OR 'brucella canis' OR 'brucella canidis' OR 'brucella ovis' OR 'brucella ovis' OR 'brucella suis' OR 'brucella melitensis biovar suis' OR 'brucella melitensisbv. suis' OR 'brucella suis'):ti,ab,kw<br>**Population:**<br>**#3** (pregnan*):ti,ab,kw<br>**Intervention:**<br>**#4** ((('therapy' OR 'combination therapy' OR 'disease therapy' OR 'disease treatment' OR 'diseases treatment' OR 'disorder treatment' OR 'disorders treatment' OR 'efficacy, therapeutic' OR 'illness treatment' OR 'medical therapy' OR 'medical treatment' OR 'multiple therapy' OR 'polytherapy' OR 'somatotherapy' OR 'therapeutic action' OR 'therapeutic efficacy' OR 'therapeutic trial' OR 'therapeutic trials' OR 'therapeutics' OR 'therapy, medical' OR 'treatment effectiveness' OR 'treatment efficacy' OR 'treatment, medical'))<br>**#5** (#1 OR #2) AND #3 AND #4	27
**BVS**	**Population**<br>**#1** ((((mh:(brucelose)) OR (brucelose) OR (mh:(brucellosis)) OR (brucellosis) OR (mh:(brucelosis)) OR (brucelosis) OR (febre ondulante) OR (febre de malta) OR (infecçãopor brucella) OR (mh:(brucella)) OR (brucella) OR (mh:(brucella abortus)) OR (brucella abortus) OR (bacterium abortus) OR (mh:(brucella canis)) OR (brucella canis) OR (mh:(brucella melitensis)) OR (brucella melitensis) OR (micrococcus melitensis) OR (mh:(brucella ovis)) OR (brucella ovis) OR (mh:(brucella suis)) OR (brucella suis))))<br>**Population**<br>**#2** (pregnan*)<br>**Intervention:**<br>**#3** ((((therapeutics OR therapeutic OR therapy OR therapies OR treatment OR treatments OR (anti-bacterial agents) OR (agents, anti-bacterial) OR (anti bacterial agents) OR (antibacterial agents) OR (agents, antibacterial) OR (antibacterial agent) OR (agent, antibacterial) OR (anti-bacterial compounds) OR (anti bacterial compounds) OR (compounds, anti-bacterial) OR (anti-bacterial agent) OR (agent, anti-bacterial) OR (anti bacterial agent) OR (anti-bacterial compound) OR (anti bacterial compound) OR (compound, anti-bacterial) OR (bacteriocidal agents) OR (agents, bacteriocidal) OR (bacteriocidal agent) OR (agent, bacteriocidal) OR (bacteriocide) OR (bacteriocides) OR (anti-mycobacterial agents) OR (agents, anti-mycobacterial) OR (anti mycobacterial agents) OR (anti-mycobacterial agent) OR (agent, anti-mycobacterial) OR (anti mycobacterial agent) OR (antimycobacterial agent) OR (agent, antimycobacterial) OR (antimycobacterial agents) OR (agents, antimycobacterial) OR (antibiotics) OR (antibiotic)))))<br>**Bases available after removing MEDLINE from the VHL filter:**<br>**#4** (db:("LILACS" OR "BINACIS" OR "IBECS"))<br>**#5** #1 AND #2 AND #3 AND #4	29
**Total**		737

Search carried out on December 12, 2023, updated on September 15, 2025.

Supplementary Table S2 - Studies excluded after full text reading

Author	Year	Title	Reason for exclusion
Dunn	2025	Brucellosis in pregnancy: a case report.	Less than 5 participants
Liu	2025	Intrauterine fetal death due to Brucella melitensis infection: a case report and literature review.	Less than 5 participants
Rastegar	2025	Brucellosis (Malta Fever) in Pregnancy and Childbirth: A Case Report	Less than 5 participants
Elizalde-Bielsa	2024	Epidemiological, clinical, biochemical, and treatment characteristics of brucellosis cases in Turkey	Population (secondary manifestations of human brucellosis)
Poveda-Urkixo	2024	Brucella melitensis Rev1Δwzm: Placental pathogenesis studies and safety in pregnant ewes	Study design (in vitro study)
Glick	2016	Brucella melitensis (bm) bacteremia in hospitalized adult patients in southern Israel.	Language
Vilchez	2015	Brucellosis in pregnancy: clinical aspects and obstetric outcomes.	Outcome*
Al-Tawfiq	2013	Pregnancy associated brucellosis.	Study design (literature review)
Gulsun	2011	Brucellosis in pregnancy.	Population (Repeated participants)
Mohammad	2011	Maternal brucellosis and human pregnancy.	Outcome*
Bagirova	2008	The particularities of complex therapy of chronic brucellosis at pregnancy	Language
Garriguet	2000	Brucelosis y gestación - Brucelosis and pregnancy	Study design (literature review)
Oscherwitz	1995	Brucellar bacteremia in pregnancy.	Less than 5 participants
Kelly	1987	Pregnancy and brucellosis.	Less than 5 participants
Quentin	1983	Abortion consecutive to a salpingitis caused by Brucella melitensis biotype 1	Language
Tauchnitz	1979	Brucellosis therapy in pregnancy	Language
Tauchnitz	1978	Treatment of brucellosis during pregnancy	Language
Bechara	1978	Human brucellosis and abortion	Study design (literature review)
Porreco	1974	Brucellosis in pregnancy	Less than 5 participants

*The reason “outcome” indicates that the article did not address any of the outcomes of interest defined a priori for this systematic review.
